# The effect of Kinesio taping on postoperative complications following mandibular third molar surgery/extraction: a systematic review and meta-analysis of randomized controlled trials

**DOI:** 10.3389/fdmed.2025.1709498

**Published:** 2025-12-19

**Authors:** Ahmed Abu-Zaid, Yousef Talal Aldoseri, Abdulwahab T. A. Alenezi, Husain A. M. Dashti, Lolwah Fahad Aldhafairi, Mohammad D. H. Oqlah, Mohammed Ibrahim Alkandari, Abdullah Mohammed Aldulaimi, Abdulaziz Salem Owayed, Sayed Ali Alsaleh, Meshari A. Alobayian, Hanan A. Alshammari

**Affiliations:** 1 Department of Biochemistry and Molecular Medicine, College of Medicine, Alfaisal University, Riyadh, Saudi Arabia; 2 Riyadh Elm University, Riyadh, Saudi Arabia; 3 Al-Jahra Hospital, Aljahra, Kuwait; 4 Ministry of Health, Kuwait City, Kuwait; 5 Faculty of Dentistry, Cairo University, Cairo, Egypt

**Keywords:** extraction, Kinesio taping, mandibular, meta-analysis, postoperative complication, surgery, systematic review, third molar

## Abstract

**Background/objectives:**

The surgical removal of mandibular third molars is frequently followed by pain, swelling, and trismus. Conventional management relies on analgesics, anti-inflammatory drugs, and corticosteroids, but interest in non-pharmacological adjuncts persists. Kinesio taping (KT) has been explored in randomized trials as a potential aid in reducing postoperative morbidity. This review aimed to evaluate the efficacy of KT after mandibular third molar surgery.

**Methods:**

Five databases were searched until September 2025. Eligible studies were randomized controlled trials (RCTs) comparing KT with standard care, no taping, or sham taping after mandibular third molar extraction, reporting pain, swelling, or maximum interincisal opening (MIO). Data were extracted independently and pooled as mean differences (MDs) with 95% confidence intervals using random-effects meta-analysis. Risk of bias was assessed using the Cochrane Risk of Bias 2 tool.

**Results:**

In total, 16 RCTs, involving 721 participants, were included. KT significantly reduced pain on day 1 (MD = −1.25, *p* < 0.001), day 3 (MD = −0.88, *p* = 0.04), and day 7 (MD = −1.08, *p* = 0.01), though not on day 2. Swelling reduction was significant on day 2 (MD = −2.45 cm, *p* < 0.001) but not on day 7. The MIO was greater in the KT group on days 2 (MD = 4.8 mm, *p* < 0.001), 3 (MD = 3.72 mm, *p* = 0.02), and 7 (MD = 2.41 mm, *p* = 0.04).

**Conclusion:**

KT appears to provide short-term benefits in pain control, swelling reduction, and mouth opening after third molar surgery. While promising, effect sizes vary, and heterogeneity underscores the need for larger, standardized RCTs to define optimal techniques and confirm clinical value.

**Systematic Review Registration:**

https://www.crd.york.ac.uk/PROSPERO/view/CRD420251144699, PROSPERO CRD420251144699.

## Introduction

The surgical removal of mandibular third molars is routine in oral and maxillofacial practice, yet many patients experience a predictable cluster of early postoperative problems (1). The most common symptoms are pain, facial swelling (edema), and reduced mouth opening (trismus) (2). Clinically, pain rises as local anesthesia wanes, swelling peaks at approximately 48 h, and mouth opening gradually recovers over the first week as the edema resolves (3). These patterns are well-documented in third molar extraction cohorts using the following standard outcome measures: pain on a 0–10 visual analog scale (VAS); trismus, using maximum interincisal opening (MIO; mm); and edema, using extra-oral linear distances or three-dimensional (3D) facial volumetry (3–5).

Our default postoperative regimen combines non-steroidal anti-inflammatory drugs (NSAIDs), paracetamol/acetaminophen, and perioperative corticosteroids when indicated to temper the inflammatory cascade and improve comfort (6). Current evidence syntheses support these choices. The literature indicates that ibuprofen and/or paracetamol provide reliable analgesia after lower third molar extraction, and that dexamethasone, when used appropriately, reduces pain, facial swelling, and trismus across a range of doses and routes (7, 8). In a Cochrane review of seven randomized trials, ibuprofen 400 mg was superior to paracetamol 1,000 mg after lower wisdom tooth removal, with a higher proportion of patients achieving at least 50% pain relief over 6 h (6). A more recent meta-analysis focusing on third molar surgery confirmed that single-dose ibuprofen 400 mg provides greater overall pain relief and similar safety when compared with acetaminophen and other traditional non-opioid analgesics (9). As clinicians, we balance these benefits against contraindications and occasional adverse effects, which motivates interest in safe, non-pharmacological adjuncts that can complement rather than replace routine care.

Kinesio taping (KT) is one such adjunct. It uses a thin, elastic, acrylic-adhesive textile applied with low tension along lymphatic pathways and over involved muscles (10). The proposed mechanisms are straightforward from a clinical standpoint: gentle skin lifting to alter interstitial pressure and facilitate lymphatic flow, together with cutaneous mechanoreceptor input that can modulate nociception (gate-control) (11). In lymphatic “fan” applications commonly adapted for the face, tails are laid from the supraclavicular region toward the masseteric/infra-zygomatic area with minimal (≈0%–20%) tension and the tape is typically left in place for several days, provided the skin is intact and patients have no adhesive allergy (12). Evidence for these mechanisms and parameters comes from broader rehabilitation literature and manufacturer protocols, and they align with our maxillofacial goals of edema control and comfort restoration (13).

Within oral surgery, randomized split-mouth and parallel-group trials have examined KT after third molar extraction, reporting effects on pain, edema, and mouth opening at early (≤48–72 h) and late (≈day 7) timepoints. Techniques vary (e.g., lymphatic fan vs. muscle-oriented patterns), and comparators include standard care, sham taping, or alternative adjuncts such as drains (14–17). This diversity reflects real-world practice but also introduces heterogeneity, particularly in how facial swelling is quantified. Many trials use linear landmark sums, whereas newer studies favor 3D stereophotogrammetry or scanning workflows that better capture volumetric change (5, 18, 19).

Two recent meta-analyses summarized this literature. One reported reduction in pain, swelling, and trismus across early and late periods; another, restricted to randomized trials, found that benefits for pain and swelling were most apparent before day 7, with consistent improvements in mouth opening. However, both highlighted small samples and between-study heterogeneity (including outcome measurement), leaving uncertainty about the size and durability of any benefit (20, 21). These gaps, together with several randomized controlled trials (RCTs) published since the most recent meta-analyses, justify an updated RCT-only synthesis focused specifically on mandibular third molar extraction surgery and allow us to leverage a larger cumulative sample size for greater precision in effect estimates (14, 15, 18, 22–34).

## Methods

### Protocol and reporting

This systematic review and meta-analysis adhered to the Preferred Reporting Items for Systematic reviews and Meta-Analyses (PRISMA) 2020 recommendations and the Cochrane Handbook for Systematic Reviews of Interventions (35, 36). Methods were specified *a priori* in a protocol that defined the review question, eligibility criteria, prespecified outcomes and timepoints, and the statistical plan. The protocol was registered on PROSPERO (CRD420251144699).

### Eligibility criteria

We included randomized controlled trials, parallel-group or split-mouth, that enrolled patients who underwent mandibular third molar extraction and compared KT to standard care without KT, no taping, or sham/placebo taping. Trials were eligible if they reported at least one of the following outcomes: postoperative pain on a 0–10 visual analog or numeric rating scale, facial swelling measured extra-orally by linear landmark methods or by 3D volumetry, or MIO as an index of trismus. Non-randomized and quasi-randomized studies were excluded, and no limits were applied regarding language, setting, or publication status within the adolescent–adult population typically treated for third molars.

### Information sources and search

We searched MEDLINE (via PubMed), Cochrane, Web of Science, Embase, and Scopus from inception to 15 September 2025, screened the reference lists of eligible studies and relevant reviews, and conducted forward citation tracking. Search strategies combined terms for third molars/wisdom teeth and kinesio/elastic therapeutic taping. Full, database-specific search strings are provided in Supplementary Table S1. No date or language filters were applied at the search stage.

### Screening and study selection

Two reviewers independently screened records in two stages (titles/abstracts, then full texts) using Rayyan with piloted eligibility forms (37). Disagreements were resolved through discussion, with arbitration by a third reviewer when required. Reasons for full-text exclusion were recorded, and the selection process is presented in a PRISMA flow diagram (Supplementary Figure S1).

### Data extraction

Two reviewers independently extracted data using a standardized template, including study design, setting and country, sample size, participant characteristics, surgical details, peri-/postoperative medications, intervention parameters for KT, and comparator details. For each outcome and timepoint, we extracted the reported group means with their standard deviations exactly as presented in the included trials and synthesized the data at the prespecified postoperative timepoints.

### Risk of bias assessment

The risk of bias was assessed independently by two reviewers using the Cochrane Risk of Bias 2 (ROB-2) tool (38) at the outcome level, covering the randomization process, deviations from intended interventions, missing outcome data, outcome measurement, and selection of the reported result. Disagreements were resolved by consensus or third reviewer adjudication.

### Certainty of evidence

To evaluate the certainty of evidence for each outcome, we applied the Grading of Recommendations Assessment, Development, and Evaluation (GRADE) approach. This framework considers the following five key domains: risk of bias, inconsistency, indirectness, imprecision, and other relevant factors. Based on these domains, we assigned an overall rating of high, moderate, low, or very low certainty of evidence.

### Outcomes

The primary outcomes were postoperative pain, facial swelling (edema), and mouth opening (trismus) following mandibular third molar extraction. Pain was assessed on a 0–10 visual analog or numeric rating scale on postoperative days 1, 2, 3, and 7. Swelling was assessed using extra-oral linear landmark measurements or 3D facial volumetry with prespecified pooling on postoperative days 2 and 7. Mouth opening was assessed as MIO on postoperative days 2, 3, and 7.

### Statistical analysis

Continuous outcomes were synthesized as mean differences with 95% confidence intervals, separately for each outcome and prespecified timepoint. Statistical heterogeneity was quantified using *I*^2^ and *τ*^2^ and evaluated with Cochran's *Q* test (*p* < 0.10 interpreted as evidence of heterogeneity; *I*^2^ > 50% considered substantial) (36). No subgroup analyses were conducted because the available evidence did not provide sufficiently detailed or consistently reported data to support reliable stratification. Influence was examined using leave-one-out analyses, and potential outliers were explored with Galbraith (radial) plots with re-estimation after exclusion when indicated. Small study effects were assessed using funnel plots and Egger's regression when at least 10 studies contributed to a given outcome and timepoint. Split-mouth trials were analyzed using paired or within-participant estimates when reported. Meta-analyses were conducted in Stata MP version 17 (StataCorp).

## Results

### Search results

The initial search across five electronic databases identified 408 records: PubMed (*n* = 23), Embase (*n* = 153), Cochrane Library (*n* = 36), Web of Science (*n* = 56), and Scopus (*n* = 140). After removing 126 duplicates, 282 unique articles remained for title and abstract screening. Of these, 266 studies were excluded in the first step of screening as they did not meet the inclusion criteria. The full texts of the remaining 16 articles were assessed for eligibility (14, 15, 18, 22–34), and all were included in the qualitative and quantitative synthesis (Supplementary Figure S1).

### Summary of included studies

Overall, 16 clinical studies met the eligibility criteria (14, 15, 18, 22–34). They evaluated the efficacy of KT for managing postoperative complications (primarily swelling, pain, and trismus) following mandibular third molar extractions. Of these, 12 were parallel RCTs and four were split-mouth RCTs. The studies were conducted across seven countries, with the majority from India (*n* = 5) and Turkey (*n* = 5). In total, the studies involved 721 participants, with the KT arms totaling 366 participants and the no-KT (control) arms totaling 355 participants. Gender distribution was generally balanced across studies, though some showed female predominance; a few studies did not report gender breakdown. The participants were mostly healthy adults aged 16–64 years, with most studies focusing on the 18–40 age range. KT was applied using different techniques, commonly from the supraclavicular lymph nodes to facial areas of expected edema, with follow-up periods ranging from 1 to 7 days. Nearly all the studies used quantitative swelling measurements (e.g., three- to five-line methods, 3D imaging. Table 1 and Supplementary Table S2.

**Table 1:** Characteristics of the included studies.

Study ID		Study design	Country	Total participants	Trial arm		Kinesio taping				Pre-medication	Post-medication	Swelling		Impaction type	Main inclusion criteria	Conclusion	Follow-up (days)
					Intervention	Control	KT type	Technique used	Size of tape	Duration the tape remained in place for			Measurement method	Form of data presentation				
1	Abhijith et al. (2025)	RCT	India	40	KT	No-KT	(NEYMA Waterproof Non-invasive Kinesiology Sports Tape)	Modified web strip technique (vertical direction) extending from line C [tragus (T) to exocanthion] to the supraclavicular region along the neck	Not reported	3 days	Not reported	Routine antibiotics and NSAIDs	Manually using a flexible measuring scale	Mea*n* ± SD	Not reported	ASA-1 patients between the ages of 18 and 40	KT, when used with anti-inflammatory medications, is a non-invasive and effective approach for managing postoperative complications	3, 7
2	Chiang et al. (2021)	RCT	India	76	KT	No-KT	Not reported	The application of KT was done from the supraclavicular region to the point of maximum swelling	Not reported	Not reported	Not reported	Analgesics and antibiotics	Means of Five-line	Mean ± SD (cm)	Slightly or moderately difficult	Healthy individual between 18 and 40 years of age with no systemic disease, history of allergy, or bleeding problem	The patients in the KT group had decreased pain, trismus, and swelling and improved quality of life compared with those in the control group	3, 5, 7
3	Gözlüklü et al. (2019)	Split-mouth RCT	Turkey	30	KT	No-KT	Skin- or black-coloured Kinesio-Tex Gold®,	Technique A: Placed just above the supraclavicular lymph nodes (the target area for drainage); Technique B: A masseteric support bandage was placed in addition to the tapes used in the classic technique A, defined by the distance (in the stretched position) between the clavicle and the position of the most severe swelling	50 mm × 5 m	5 days	Not reported	Amoxicillin + clavulanate (1 g) + Naproxen sodium (500 mg) + chlorhexidine gluconate MW	3dMD Face System	Scale 0–100 Mean ± SD	Class I position B and C	Healthy individual aged over 18 years, compliant of bilateral mandibular wisdom tooth impaction	KT is a useful method for reducing postoperative morbidity following impacted third molar extraction	2, 7
4	Heras et al. (2020)	Split-mouth RCT	Brazil	26	KT	No-KT	Beige tape application (Leukotape KeBNS)	Started from the base of the mandible in the submandibular ganglion chain region (fixed point) and covered the area below the ear lobe towards the entire labial commissure extension	50 mm × 5 m	5 days	Dexamethasone (4 mg), Amoxicillin (2 g)	Paracetamol (750 mg) Dexamethasone (4 mg) Amoxicillin (500 mg) Chlorhexidine MW (0.12%)	Distance between the mentum apex and the lowest part of the ear lobe	Median (1°–3° Quartile)	Position C	Healthy individual older than 18 years, asymptomatic bilateral and impacted mandibular third molar in a mesioangular (Winter classification) position (Pell and Gregory classification: class C)	KT was effective in reducing swelling and pain	2, 5
5	Jaron et al. (2021)	RCT	Poland	100	KT	No-KT	K-Active Tape Classical	The application of the tape was started in the area of the supraclavicular lymph nodes. The tape was then advanced to line A (tragus to cheilion) on the patient’s face where the greatest edema was expected	50 mm × 5 m	5 days	Not reported	Ketoprofen (100 mg) chlorhexidine solution (0.1%)	Five-line mapped	Mean ± SD	Not reported	Caucasian patients older than 18 years, asymptomatic impacted mandibular third molar	KT was effective in reducing postoperative edema, pain, and trismus after impacted mandibular wisdom tooth surgery	3, 7
6	Kim et al. (2020)	RCT	South Korea	40	KT	No-KT	Skin-colored Nitto Kinesio Tape	The base was placed above the area drained by the supraclavicular nodes. The tape placement was directed towards the appropriate lymphatic ducts crossing the cervical, submental, submandibular, and parotid nodes	50 mm × 5 m	Not reported	Not reported	Intravenous tramadol (50 mg/mL), Intravenous Ceftriaxone sodium hydrate (1 g) Chlorhexidine (0.12%)	Four-line measurement method using a standard plastic tape placed in contact with the skin	Mean ± SD (cm)	1, 2, 3	Dentigerous cyst with mandibular third molar extraction	KT can effectively manage facial swelling after oral and maxillofacial surgeries such as cyst enucleation and third molar extraction	3
7	Menziletoglu et al. (2020)	RCT	Turkey	60	KT	No-KT	Kinesiology Tape Nill Flex	Tapes (1.6 cm in width) were applied between the tragus-commissure and the clavicle and the base of the three strips was placed just above the supraclavicular nodes	50 mm × 5 cm	2 days	Not reported	Amoxicillin, paracetamol (500 mg), Benzydamine HCl + chlorhexidine gluconate MW	Three-line measurement using a ruler measure	Mean ± SD	Class II position B	Patient between the ages 18–40 years, mesioangular impacted mandibular third molars (Winter classification, Pell and Gregory class II, position B), fully covered with bone and mucosa, and no medication use	Regarding pain and swelling, the effects of a drainage tube and KT were similar to those of the control	2, 7
8	Narayan et al. (2025)	RCT	India	40	KT	No-KT	K-Active Tape Classical	The application of the tape started from the area of the supraclavicular lymph nodes. The tape is then advanced to the ala-tragal line on the patient’s face, where the greatest edema is expected	200 mm×5 m	3 days	Not reported	Antibiotics and analgesics (without serratiopeptidase)	Five-line measurement using a flexible scale	Mean ± SD	Not reported	Patients who were willing to participate in the study, aged between 17 and 55 years	KT showed significant improvement in trismus and swelling on postoperative days 3 and 7 in the study group	3, 7
9	Patil et al. (2023)	Split-mouth RCT	India	15	KT	No-KT	Kinesio Tex Gold Finger Print	Placing the KT between the clavicle and the tragus commissure line on the patient’s face	50 mm×5 m	7 days	Not reported	Not reported	Three-line measurement	Mean ± SD	Not reported	Healthy individual aged over 18 years, with no history of pathological condition or any pharmacological therapy	KT enables patients to have a comfortable time post-operatively and helps to regain a better quality of life	1, 2, 3, 7
10	Pławecki et al. (2023)	RCT	Poland	30	KT	No-KT	Kinesio Tex Classic tape	The skin surfaces on the side of the procedure were covered, starting from the supraclavicular fossa, through the mandible, and ending with the swollen buccal area	50 mm×4 m	3–5 days	Not reported	100 mg of ketoprofen, Ketonal Forte, in case of pain as needed	Five distances (in mm) were measured on the basis of six reference points on the face from the angle of the mandible	Mean ± SD	Ganss ratio (A, B, C)	Patient aged between 16 and 64 years	Kinesio taping, in addition to NSAIDs was found to be more effective than NSAIDs alone in increasing the degree of jaw opening, decreasing pain intensity, and reducing the non-steroid anti-inflammatory dosage in patients after impacted mandibular wisdom teeth surgery	2, 7
11	Ristow et al. (2014)	RCT	Germany	40	KT	No-KT	Skin colored K Active Tape Classic®	The base of three strips was placed above the supraclavicular nodes. Placement of the lymphatic strips was directed by the location of the lymphatic duct, crossing the cervical, submental, mandibular, submandibular, preauricular, and parotid nodes to the area of maximum swelling	50 mm × 5 m	5 days	Ampicillin/Sulbactam	Ice pack, analgesic and anti-inflammatory medication (diclofenac, 50 mg)	Five-line measurement	Mean ± SD (cm)	Class II position B and C	Healthy patients older than 18 years, bilateral and impacted 3Ms Pell and Gregory classification: class B and C	KT offers patients a less traumatic postoperative experience and, therefore, holds promise to enhance the quality of life of a large cohort of the population	1, 2, 3, 7
12	Russo et al. (2025)	Split-mouth RCT	Italy	7 (14 surgical procedures)	KT	No-KT	Thin cotton layer with an acrylic adhesive (latex-free) applied in a wave pattern	Lymphatic correction technique	50 × 5 m	7 days	Not reported	Antibiotics and analgesics	Smartphone (iPhone X, iOS 16.7.6) with the integrated TrueDepth front camera and a third-party scanner application available in the Apple App Store (Heges 3D, version 1.7, Marek Simonik)	Volume	Class I–II position A-B	Patient aged between 17 and 40 years, having both mandibular third molars impacted and symmetrical, located in a mesio-angular or horizontal position	KT proved to be a safe and effective method for improving postoperative recovery following mandibular third molar surgery, offering a low-cost, accessible option to enhance patient comfort and quality of life	3, 7
13	Tatli et al. (2020)	RCT	Turkey	40	KT	No-KT	Kinesio® Tex GoldTM	The taping material was cut into five pieces. Then, the base of the five-strip taping material was applied slightly above the supraclavicular lymph nodes without tension	Not reported	5 days	Not reported	Amoxicillin + clavulanate, Flurbiprofen, benzydamine HCl + chlorhexidine gluconate MW	Three-line measurement using a flexible plastic tape measure	Mean ± SD (cm)	Class II position B	Healthy patients older than 18 years, impacted mandibular third molar class II position B (Pell and Gregory classification)	KT was effective in reducing morbidity after impacted mandibular third molar surgery	2, 4, 7
14	Teke et al. (2025)	RCT	Turkey	87	KT	No-KT	Kinesio-tex gold tapes	Lymphatic drainage technique with 60% tension	Not reported	7 days	Not reported	Amoxicillin + clavulanic acid (875 mg + 125 mg), paracetamol (500 mg)	Five-distances	Mean ± SD (cm)	Class II position B	Healthy patients between the ages of 18 and 30 years, only if they had a single mandibular third molar extraction	KT application was associated with increased blood flow and minimized postoperative pain, edema, and trismus	2, 4, 7
15	Tusharbhai et al. (2020)	RCT	India	30	KT	No-KT		Extending from the supraclavicular to the position of highest swelling		5 days	Not reported	Not reported	Six definite time intervals	Mean ± SD (cm)	Position B and C	Individuals aged 18 years and above with bilateral maxillary and mandibular impacted third molars	KT is a self-effacing, less traumatic, economical approach, which is free from adverse reactions and improves patients’ quality of life	1, 2, 3, 5
16	Yurttutan et al. (2020)	Split-mouth RCT	Turkey	60	KT	No-KT	Skin-colored Kinesio Tex Gold	Tapes were applied to the masseteric region where the most severe edema was observed and the measurements were performed. The web strip method (in which the tape has solid ends and four longitudinal cuts through the center section) was applied	50 mm × 5 m	7 days	Not reported	Analgesic (not specified)	Three-line measurement using a flexible plastic tape measure	Mean ± SD (mm)	Class I–II position B	Healthy patients between the ages of 18 and 35 years with no history of facial trauma, no other medical conditions, no pericoronitis or pain before surgery, and bilateral, symmetric, impacted lower 3Ms (Pell and Gregory classification: class I-B and II-B).	KT with the web strip technique was more economical and less traumatic than KT with other approaches	1, 2, 3, 7

### Summary of quality assessment and certainty of evidence

Supplementary Figure S2 summarizes the quality assessment of the included studies. Overall, the quality varied, with 10 studies judged as having some concerns, five as low risk of bias, and one as high risk of bias. The majority of the studies had some concerns in Domain 1, primarily due to insufficient details regarding the randomization and allocation processes. One study (29) was judged to have a high risk of bias in Domain 1 because allocation was not concealed; the researchers assigned interventions using known even and odd numbers. One study (24) was judged as having some concerns in Domain 5 because the measure of variability (i.e., whether a range or interquartile range was used) was not clearly specified, leading to a lack of clarity when analyzing the postoperative pain outcome. Another study (31) was judged as having some concerns in Domain “5” because baseline data were not reported, making it impossible to include the pain outcome in the meta-analysis. The GRADE assessment showed that the certainty of evidence varied across outcomes. Postoperative pain on days 1 and 2 and trismus on day 2 were rated moderate quality, downgraded due to serious risk of bias but without major issues in inconsistency, indirectness, or imprecision. In contrast, pain on days 3 and 7, swelling on days 2 and 3, and trismus on days 3 and 7 were all judged low quality because of serious risk of bias and significant heterogeneity. No additional concerns, such as publication bias or confounding, were identified. Supplementary Table S3 summarizes the overall GRADE findings.

### Meta-analysis

#### Meta-analysis of postoperative pain VAS scores

The KT group demonstrated significantly lower postoperative pain VAS scores compared to the no-KT group on day 1 (*n* = 6 studies, MD = −1.25, 95% CI [−1.72, −0.79], *p* < 0.001), day 3 (*n* = 11 studies, MD = −0.88, 95% CI [−1.72, −0.03], *p* = 0.04), and day 7 (*n* = 11 studies, MD = −1.08, 95% CI [−1.89, −0.27], *p* = 0.01). There was no significant difference between the groups on postoperative day 2 (*n* = 9, MD = 1.21, 95% CI [−1.76, 4.18], *p* = 0.42) (Supplementary Figure S3). Pooled analyses showed low heterogeneity across all timepoints (*I*^2^ < 50%, *p* > 0.1), except for postoperative days 3 (*I*^2^ = 94.78%, *p* < 0.001) and 7 (*I*^2^ = 98.67%, *p* < 0.001), which exhibited substantial heterogeneity (Supplementary Figure S3). Galbraith plots identified the study by Abhijith et al. (22) as a potential outlier for postoperative day 3. No outliers were detected for postoperative day 7 (Supplementary Figure S4). The sensitivity analysis (leave-one-out) indicated that the results for all timepoints were robust, except for postoperative day 2, where exclusion of certain individual studies altered the overall effect size and rendered the result statistically insignificant (Supplementary Figure S5). The funnel plots for postoperative days 3 and 7 showed asymmetry, suggesting potential bias. However, Egger's regression test did not indicate statistical significance at either timepoint (*p* = 0.6559 and *p* = 0.6647, respectively) (Supplementary Figure S6).

#### Meta-analysis of postoperative swelling

The KT group exhibited significantly reduced swelling compared to the no-KT group on postoperative day 2 (*n* = 8 studies, MD = −2.45 cm, 95% CI [−3.79, −1.10], *p* < 0.001), but not on day 7 (*n* = 8 studies, MD = −0.09 cm, 95% CI [−0.48, 0.29], *p* = 0.64) (Supplementary Figure S7). Pooled analyses revealed significant heterogeneity at all timepoints (*I*^2^ > 50%, *p* < 0.1) (Supplementary Figure S7). Galbraith plots did not identify any potential outliers (Supplementary Figure S8). The sensitivity analysis (leave-one-out) demonstrated the robustness of the results, as the exclusion of individual studies did not materially affect the overall effect size (Supplementary Figure S9).

#### Meta-analysis of postoperative trismus (mouth opening)

The KT group exhibited significantly greater trismus compared to the no-KT group on postoperative day 2 (*n* = 6 studies, MD = 4.8 cm, 95% CI [3.15, 6.45], *p* < 0.001), day 3 (*n* = 6 studies, MD = 3.72 cm, 95% CI [0.72, 6.71], *p* = 0.02), and day 7 (*n* = 9 studies, MD = 2.41 cm, 95% CI [0.17, 4.65], *p* = 0.04) (Supplementary Figure S10).

Pooled analyses revealed substantial heterogeneity across all timepoints (*I*^2^ > 50%, *p* < 0.1) (Supplementary Figure S10). Galbraith plots identified the study by Tatli et al. (18) as a potential outlier at day 2, whereas the study by Abhijith et al. (22) was identified as an outlier at both day 3 and day 7 (Supplementary Figure S11).

The sensitivity analysis using the leave-one-out method demonstrated that the findings for day 2 were robust. However, for days 3 and 7, the exclusion of individual studies significantly altered the overall effect size and rendered the results statistically non-significant (Supplementary Figure S12).

## Discussion

Across 16 RCTs (12 parallel and four split-mouth) from seven countries and 721 participants (366 KT; 355 controls), KT was associated with lower postoperative pain on days 1 (six studies), 3 (11 studies), and 7 (11 studies), with no consistent difference on day 2 (nine studies). For swelling, a benefit was evident on day 2 (eight studies) but not on day 7 (eight studies). For mouth opening, KT resulted in greater maximum interincisal opening on days 2 (six studies), 3 (six studies), and 7 (nine studies). Heterogeneity across trials was notable, with individual outliers influencing some timepoints. The day 2 effects were generally robust, whereas some later effects were sensitive to the exclusion of specific studies. The risk of bias assessments varied across the evidence base (five were low risk, 10 had some concerns, and one was high risk), and reporting limitations in a few trials restricted the inclusion of certain outcomes.

In comparison with previous meta-analyses (20, 21), our findings reinforce and extend the available evidence that Kinesio taping mitigates key postoperative complications after mandibular third molar extraction surgery. Wang et al. reported that KT confers modest but consistent reductions in early postoperative pain, swelling, and trismus, while Firoozi et al. concluded that KT is particularly effective in lowering pain within the first 48 h and improving mouth opening across postoperative intervals, with no clear benefit for pain or swelling by day 7 (20, 21). Our findings partially align with those of Qi et al., who also reported significant reductions in postoperative pain and edema with KT (39). However, their review, based on eight studies, found no meaningful improvement in mouth opening or swelling compared with controls, whereas our larger dataset demonstrated clear benefits in early swelling reduction and sustained gains in maximum interincisal opening across postoperative days 2–7. The limitations highlighted previously, including small samples and heterogeneous measurement methods, align with the heterogeneity we observed and frame the interpretation of our pooled effects. Our analysis, furthermore, showed that the certainty of evidence ranged from moderate to low across outcomes. Early postoperative pain (days 1–2) and trismus at day 2 were supported by moderate-quality evidence, primarily limited by risk of bias. In contrast, later pain, swelling, and trismus outcomes were rated low certainty owing to combined concerns of bias and heterogeneity, with no additional downgrading for imprecision, indirectness, or publication bias. The observed early and later pain reductions with KT are consistent with expected third molar healing. Pain signaling peaks soon after surgery, and inflammation and protective muscle spasm then decline over the following days. KT may attenuate nociception by providing sustained cutaneous afferent input consistent with gate-control mechanisms, and by promoting lymphatic clearance that lowers interstitial pressure and tissue tension as edema resolves. These mechanisms are described across rehabilitation literature and align with the tape's elastic, low-tension application. Comparable edema-related benefits have been reported in postoperative and lymphatic conditions, and oral surgery trials commonly adopt lymphatic “fan” patterns to target facial swelling after third molar extraction (40–43).

In routine third molar care, effective non-opioid analgesia (for example, ibuprofen alone or in combination with acetaminophen) can narrow between-group differences on certain interim days, which helps explain the absence of a consistent effect at the second postoperative assessment despite benefits at earlier and later timepoints (44, 45). The early reduction in swelling is consistent with the natural edema trajectory after mandibular third molar extraction, where facial swelling typically peaks around 48–72 h before beginning to resolve. KT's lymphatic “fan” applications aim to facilitate fluid clearance by gently lifting the skin and altering interstitial pressure gradients, so the effect is most evident at or near the edema peak (46).

The lack of a sustained difference at the subsequent assessment likely reflects both the natural resolution phase of swelling and methodological heterogeneity across trials, including variation in how edema was quantified (linear landmark sums vs. 3D volumetry), tape wear time and reapplication, and concomitant medications (5, 47–49).

Improvements in mouth opening through the first postoperative week plausibly follow from reduced edema and analgesia, as less soft tissue pressure and pain translate to less muscle spasm and greater maximum interincisal distance. The sensitivity of the later timepoint effects to individual studies can arise from differences in rehabilitation advice (e.g., jaw exercises), baseline impaction difficulty and operative time, and KT parameters (application pattern, tension, timing, and wear duration). These sources of clinical and methodological diversity are well-recognized in third molar extraction research and studies on KT (30, 50).

The observed heterogeneity across outcomes was consistent with several factors documented in the literature, namely, variability in randomization and allocation reporting that can bias effects, diversity in KT techniques (lymphatic vs. muscle-focused patterns), inconsistent edema measurement methods (linear distances vs. validated 3D scans), and co-interventions, such as perioperative NSAIDs or steroids, that differentially influence pain and swelling. Together, these elements may produce divergent results across timepoints and studies, even when the underlying direction of the effect is similar (45, 48, 49).

Finally, the overall results from individual oral surgery RCTs that evaluate elastic therapeutic tape after third molar extraction support the direction of our pooled findings, with reduced early pain and swelling and concomitant improvements in trismus, and also illustrate how protocol nuances can affect the magnitude.

### Clinical implications

Current guidance for third molar extraction surgery favors multimodal, non-opioid analgesia as the first-line treatment, typically an NSAID alone or together with acetaminophen, and in some pathways, a single perioperative dose of dexamethasone is endorsed to lessen postoperative pain, swelling, and trismus. Routine supportive measures such as cryotherapy and standard postoperative instructions are commonly advised. Notably, major oral surgery and dental pain guidelines do not currently address KT (51–53).

Within this context, our results suggest that adding KT to existing guideline-based care reduces early pain, lessens swelling around the expected edema peak, and improves mouth opening during the first postoperative week. In practice, clinicians should consider offering KT as a non-drug adjunct for suitable patients, especially those wishing to minimize medication exposure or with contraindications to steroids or specific analgesics. Because the benefits are the clearest at the early stage, application should begin soon after surgery using a simple, reproducible lymphatic fan pattern and remain in place for several days if the skin is intact and the tape is well-tolerated. This approach is consistent with broader rehabilitation practice where taping is used as an adjunct for edema control (51).

Implementation requires dental staff training, proper skin preparation with allergy checks, and clear patient instructions regarding monitoring and tape removal. Given the heterogeneity found across trials, standardizing the taping technique, tension, and wear time would likely improve the consistency of outcomes. Importantly, KT should complement pharmacological therapy and perioperative steroids when indicated, as it is not a first-line replacement. Tape application in postoperative pathways may enhance early comfort, support faster recovery of mouth opening, and provide a low-cost, low-risk option alongside established guideline-based care.

### Strengths, limitations, and recommendations

This review only focused on RCTs, which strengthens its internal validity compared with mixed-design reviews. Additionally, the outcomes and timepoints were prespecified to mirror the clinical trajectory after third molar extraction surgery, and split-mouth trials were handled using appropriate within-participant methods when available. We examined robustness using leave-one-out analyses and explored heterogeneity using Galbraith plots, which helped identify influential studies and verify the stability of the key findings. However, we had some limitations. Heterogeneity was notable between the studies. Operation time was only reported in a few studies. The trials differed in KT technique, timing, tension, and wear duration, and in co-interventions such as NSAIDs and perioperative steroids. Swelling was measured using different approaches, including linear landmark methods and 3D imaging, which are not directly interchangeable. Some effects at later timepoints were sensitive to the exclusion of individual studies identified as outliers. Risk of bias judgments varied across trials, most commonly due to limited reporting of randomization and allocation procedures, and reporting gaps in a few studies restricted the inclusion of certain outcomes. Follow-up was short and typically limited to the first postoperative week, so longer-term effects could not be evaluated. As a result, the evidence supports KT as a practical, low-risk complement to guideline-based care, with the clearest benefits early in the postoperative period. Greater consistency is likely if application parameters such as pattern, tension, and wear duration are standardized and if co-interventions are fully documented. As a result, future RCTs are needed with standardized outcomes and assessment timepoints, ideally adding validated 3D swelling measures and follow-up beyond 1 week with clear reporting of baseline characteristics, variability, analgesic use, adverse events, and costs to enhance their clinical relevance.

## Conclusion

This study showed that KT after mandibular third molar extraction surgery was associated with less early pain, reduced swelling near the edema peak, and greater mouth opening during the first postoperative week. Effects beyond the early period were less consistent, reflecting heterogeneity in taping technique, co-interventions, and outcome measurement. KT should be considered an addition to guideline-based care rather than a replacement for established analgesic and steroid regimens.

## Data Availability

The original contributions presented in the study are included in the article/Supplementary Material, further inquiries can be directed to the corresponding author.
