# Supplementary material for: The effect of Kinesio taping on postoperative complications following mandibular third molar surgery/extraction: a systematic review and meta-analysis of randomized controlled trials
Source: Front Dent Med. 2025 Dec 19;6:1709498. doi: 10.3389/fdmed.2025.1709498 (PMC12757239; doi:10.3389/fdmed.2025.1709498)
Supplement: Supplementary file 1 [file Table1.docx]

| **Study ID** | | **Study Design** | **Country** | **Total Participants** | **Trial Arm** | | **Kinesio Taping** | | | | **Pre-Medication** | **Post-Medication** | **Swelling** | | **Impcation type** | **Main inclusion criteria** | **Conclusion** | **Follow-up (Days)** |
| --- | --- | --- | --- | --- | --- | --- | --- | --- | --- | --- | --- | --- | --- | --- | --- | --- | --- | --- |
|  |  |  |  |  | **Intervention** | **Control** | **KT type** | **Technique used** | **Size of Tape** | **Tapes remained in place for** |  |  | **Measurement methods** | **Form of data presentation** |  |  |  |  |
| **1** | **Abhijith et al. 2025** | RCT | Indian | 40 | KT | No-KT | (NEYMA Waterproof Non-invasive Kinesiology Sports Tape) | Modified Web Strip Technique (vertical direction) extending from Line C (Tragus (T) to exocanthion) to the supraclavicular region along the neck. | Not reported | 3 Days | Not reported | Routine antibiotics and NSAIDs | Manually using a flexible measuring scale | Mean ± SD | Not Reported | ASA-1 patients between the ages of 18 and 40 | KT, when used with anti-inflammatory medications, is a non-invasive and effective approach for managing postoperative complications. | 3, 7 |
| **2** | **Chiang et al. 2021** | RCT | Indian | 76 | KT | No-KT | Not reported | The application of KT was done from supraclavicular region to the point of maximum swelling | Not reported | Not reported | Not reported | Analgesics and antibiotics | Means of Five-line | Mean ± SD (cm) | Slightly or Moderately difficult | Healthy individual between 18 and 40 years of age with no systemic disease, history of allergy, or bleeding problem | The patients in the KT group had decreased pain, trismus, and swelling and improved quality of life compared with those in the control group | 3, 5, 7 |
| **3** | **Gözlüklü et al. 2019** | Split-mouth RCT | Turkey | 30 | KT | No-KT | skin- or black-coloured Kinesio-Tex Gold®, | Technique A: Placed just above the supraclavicular lymph nodes (the target area for drainage), Technique B: A masseteric support bandage was placed in addition to the tapes used in the classic technique A Define by the distance (in the stretched position) between the clavicle and the position of the most severe swelling | 50 mm × 5 m | 5 days | Not reported | Amoxicillin + clavulanate (1 g) + Naproxen sodium (500 mg) + Chlorhexidine gluconate MW | 3dMD Face System | Scale 0 to 100 Mean ± SD | Class I position B and C | Healthy individual aged over 18 years, compliant of bilateral mandibular wisdom tooth impaction | KT is a useful method for reducing postoperative morbidity following impacted third molar extraction. | 2, 7 |
| **4** | **Heras et al. 2020** | Split-mouth RCT | Brazil | 26 | KT | No-KT | Beige tape application (Leukotape KeBNS) | Started from the basis of the mandible in the submandibular ganglion chain region (fixed point), where strips were and covered the area below the ear lobe, towards the entire labial commissure extension. | 50 mm × 5 m | 5 days | Dexamethasone (4 mg), Amoxicillin (2 g) | Paracetamol (750 mg) Dexamethasone (4 mg) Amoxicillin (500 mg) Chlorhexidine MW (0.12%) | Distance between the mentum apex and the lowest part of the ear lobe | Median (1°–3° Quartile) | Position C | Healthy individual older than 18 years, Asymptomatic bilateral and impacted mandibular third molar in mesioangular (Winter classification) position (Pell and Gregory classification: class C) | KT was effective for reducing swelling and pain | 2, 5 |
| **5** | **Jaron et al. 2021** | RCT | Poland | 100 | KT | No-KT | K-Active Tape Classical | The application of the tape was started in the area of supraclavicular lymph nodes. The tape was then advanced to line A (Tragus to cheilion) on the patient’s face where the greatest edema was expected | 50 mm × 5 m | 5 days | Not reported | Ketoprofen (100 mg) Chlorhexidine solution (0.1%) | Five-line mapped | Mean ± SD | Not Reported | Caucasian patients older than 18 years, Asymptomatic impacted mandibular third molar. | KT was effective for reducing postoperative edema, pain, and trismus after impacted mandibular wisdom tooth surgery | 3, 7 |
| **6** | **Kim et al. 2020** | RCT | South Korea | 40 | KT | No-KT | Skin-colored Nitto Kinesio Tape | The base was placed above the area drained by the supraclavicular nodes. The tape placement was directed at the appropriate lymphatic ducts crossing the cervical, submental, submandibular, and parotid nodes | 50 mm × 5 m | Not reported | Not reported | Intravenous tramadol (50 mg/ml), Intravenous Ceftriaxone sodium hydrate (1 g) Chlorhexidine (0.12%) | Four-line measurement method using a standard plastic tape placed in contact with the skin. | Mean ± SD (cm) | 1, 2, 3 | Dentigerous cyst with mandibular third molar extraction | KT can effectively manage facial swelling after oral and maxillofacial surgeries such as cyst enucleation and third molar extraction, | 3 |
| **7** | **Menziletoglu et al. 2020** | RCT | Turkey | 60 | KT | No-KT | Kinesiology Tape Nill Flex | Tapes (1.6 cm in width) were applied between the tragus-commissure and the clavicle and the base of the three strips was placed just above the supraclavicular nodes | 50 mm × 5 cm | 2 days | Not reported | Amoxicillin, paracetamol (500 mg), Benzydamine HCl + chlorhexidine gluconate MW | Three-line measurement using a ruler measure | Mean ± SD | Class II position B | Patient between the ages 18 - 40 years, mesioangular impacted mandibular third molars (Winter classification, Pell and Gregory class II, position B), fully covered with bone and mucosa, no medication use | Regarding pain and swelling, the effects of a drainage tube and KT were similar to those of the control | 2, 7 |
| **8** | **Narayan et al. 2025** | RCT | Indian | 40 | KT | No-KT | K-Active Tape Classical | The application of the tape started from the area of supraclavicular lymph nodes. The tape is then advanced to the ala-tragal line on the patient’s face, where the greatest oedema is expected. | 200 mm x 5 m | 3 days | Not reported | Antibiotics and analgesics (without Serratiopeptidase) | Five-line measurement using a flexible scale | Mean ± SD | Not Reported | patients who are willing to participate in the study, age between 17 - 55 years | KT showed significant improvement in trismus and swelling on postoperative day 3 and 7 in the study group. | 3, 7 |
| **9** | **Patil et al. 2023** | Split-mouth RCT | Indian | 15 | KT | No-KT | Kinesio Tex Gold Finger Print | Placing the KT between the clavicle and the tragus commissure line on patient face | 50 mm x 5 m | 7 days | Not reported | Not reported | Three-line measurement | Mean ± SD | Not Reported | Healthy individual aged over 18 years, with no history of pathological condition or any pharmacological therapy | KT enables patients to have a comfortable time post-operatively and helps to regain better quality of life. | 1, 2, 3, 7 |
| **10** | **Pławecki et al. 2023** | RCT | Poland | 30 | KT | No-KT | Kinesio Tex Classic tape | The skin surfaces on the side of the procedure were covered, starting from the supraclavicular fossa, through the mandible, and ending with the swollen buccal area. | 50 mm x 4 m | 3 - 5 days | Not reported | 100 mg of Ketoprofenum, Ketonal Forte in case of pain as needed | Five distances (in mm) were measured on the basis of six reference points on the face from the angle of the mandible | Mean ± SD | Ganss ratio (A, B, C) | Patient aged between 16 - 64 years | Kinesio taping in addition to NSAIDs was found to be more effective than NSAIDs alone in increasing the degree of jaw opening, decreasing pain intensity, and reducing the non-steroid anti-inflammatory dosage in patients after impacted mandibular wisdom teeth surgery. | 2, 7 |
| **11** | **Ristow et al. 2014** | RCT | Germany | 40 | KT | No-KT | Skin colored K Active Tape Classic® | The base of three strips was placed above the supracla- vicular nodes Placement of the lymphatic strips was directed by the location of the lym- phatic duct crossing the cervical, sub- mental, mandibular, submandibular, preauricular, and parotid nodes to the area of maximum swelling | 50 mm × 5 m | 5 days | Ampicillin/Sulbactam | Ice pack Analgesic and Anti-inflammatory medication (Diclofenac, 50 mg) | Five-line measurement | Mean ± SD (cm) | Class II position B and C | Healthy patients older than 18 years, Bilateral and impacted 3Ms Pell and Gregory classifica- tion: class B and C | KT offers patients a less traumatic postoperative experience and, therefore, holds promise to enhance the quality of life of a large cohort of the population | 1, 2, 3, 7 |
| **12** | **Russo et al. 2025** | Split-mouth RCT | Italy | 7 (14 surgical procedures) | KT | No-KT | Thin cotton layer with an acrylic adhesive (latex-free) applied in a wave pattern. | lymphatic correction technique | 50 x 5 m | 7 days | Not reported | Antibiotics and analgesics | Smartphone (iPhone X, iOS 16.7.6) with the integrated TrueDepth front camera and a third- party scanner application available in the Apple App Store (Heges 3D, version 1.7, Marek Simonik). | Volume | Class I-II position A-B | Patient aged between 17 - 40 years, having both mandibular third molars impacted and symmetrical, located in a mesio-angular or horizontal position | KT proved to be a safe and effective method for improving postoperative recovery following mandibular third molar surgery, offering a low-cost, accessible option to enhance patient comfort and quality of life. | 3, 7 |
| **13** | **Tatli et al. 2020** | RCT | Turkey | 40 | KT | No-KT | Kinesio® Tex GoldTM | The taping material was cut into five pieces then the base of the five-strip taping material was applied slightly above the supracla- vicular lymph nodes without tension | Not reported | 5 days | Not reported | Amoxicillin + clavulanate Flurbiprofen benzydamine HCl + chlorhexidine gluconate MW | Three-line measurement using a flexible plastic tape measure | Mean ± SD (cm) | Class II position B | Healthy patients older than 18 years, Impacted mandibular third molar class II position B (Pell and Gregory classifica- tion) | KT was effective for reducing morbidity after impacted mandibular third molar surgery | 2, 4, 7 |
| **14** | **Teke et al. 2025** | RCT | Turkey | 87 | KT | No-KT | Kinesio-tex gold tapes | Lymphatic drainage technique with 60% tension | Not reported | 7 days | Not reported | Amoxicillin + clavulanic acid (875 mg + 125 mg), Paracetamol (500 mg) | Five-distances | Mean ± SD (cm) | Class II position B | Healthy patients between the ages of 18-30 years, only if they had a single mandibular third molar extraction. | KT application was associated with increased blood flow and minimized postoperative pain, edema, and trismus. | 2, 4, 7 |
| **15** | **Tusharbhai et al. 2020** | RCT | Indian | 30 | KT | No-KT |  | Extending from the supraclavicular to the position of highest swelling. |  | 5 days | Not reported | Not reported | Six definite time intervals | Mean ± SD (cm) | Position B and C | Individual aged 18 years and above with the existence of maxillary and mandibular impacted third molars bilaterally | KT is a self-effacing, less traumatic, economical approach, which is free from an adverse reaction and improves patients’ quality of life. | 1, 2, 3, 5 |
| **16** | **Yurttutan et al. 2020** | Split-mouth RCT | Turkey | 60 | KT | No-KT | Skin-colored Kinesio Tex Gold | Tapes were applied to the masseteric region, that the most severe edema was observed and the measurements were performed The web strip method (where the tape has solid ends and four longitudinal cuts through the center section) was applied | 50 mm × 5 m | 7 days | Not reported | Analgesic (not specified) | Three-line measurement using a flexible plastic tape measure | Mean ± SD (mm) | Class I-II position B | Healthy patients between the ages of 18 and 35 years with no history of facial trauma, no other medical conditions, no peric- oronitis or pain before surgery, Bilateral, symmetric, impacted lower 3Ms (Pell and Gregory classifica- tion: class I-B and II-B) | KT with the web strip technique was more economical and less trau- matic than KT with other approaches | 1, 2, 3, 7 |

**Table 1:** Characteristics of the included studies.
